# Acquired immune responses to three malaria vaccine candidates and their relationship to invasion inhibition in two populations naturally exposed to malaria

**DOI:** 10.1186/s12936-016-1112-1

**Published:** 2016-02-05

**Authors:** Otchere Addai-Mensah, Melanie Seidel, Nafiu Amidu, Dominika J. Maskus, Stephanie Kapelski, Gudrun Breuer, Carmen Franken, Ellis Owusu-Dabo, Margaret Frempong, Raphaël Rakotozandrindrainy, Helga Schinkel, Andreas Reimann, Torsten Klockenbring, Stefan Barth, Rainer Fischer, Rolf Fendel

**Affiliations:** Fraunhofer Institute for Molecular Biology and Applied Ecology IME, Forckenbeckstraße 6, 52074 Aachen, Germany; RWTH Aachen University, Institute for Molecular Biotechnology, Worringerweg 1, 52074 Aachen, Germany; Faculty of Allied Health Sciences, Kwame Nkrumah University of Science and Technology, Kumasi, Ghana; Department of Biomedical Laboratory Science, School of Medicine and Health Science, University for Development Studies, Tamale, Ghana; Kumasi Centre for Collaborative Research, Kwame Nkrumah University of Science and Technology, Kumasi, Ghana; Department of Molecular Medicine, Kwame Nkrumah University of Science and Technology, Kumasi, Ghana; Laboratoire de Microbiologie et de Parasitologie, ESSAGRO-Faculté de Médecine, Université d’Antananarivo, Antananarivo, Madagascar; Department of Experimental Medicine and Immunotherapy, Institute for Applied Medical Engineering at RWTH Aachen University and Hospital, Pauwelsstraße 20, 52074 Aachen, Germany; South African Research Chair in Cancer Biotechnology, Institute of Infectious Disease and Molecular Medicine (IDM), Department of Integrative Biomedical Sciences, Faculty of Health Sciences, University of Cape Town, Anzio Road, Observatory, 7925 South Africa

**Keywords:** *Plasmodium falciparum*, Immune response, ELISA, Invasion inhibition

## Abstract

**Background:**

Malaria still represents a major cause of morbidity and mortality predominantly in several developing countries, and remains a priority in many public health programmes. Despite the enormous gains made in control and prevention the development of an effective vaccine represents a persisting challenge. Although several parasite antigens including pre-erythrocytic antigens and blood stage antigens have been thoroughly investigated, the identification of solid immune correlates of protection against infection by *Plasmodium falciparum* or clinical malaria remains a major hurdle. In this study, an immuno-epidemiological survey was carried out between two populations naturally exposed to *P. falciparum* malaria to determine the immune correlates of protection.

**Methods:**

Plasma samples of immune adults from two countries (Ghana and Madagascar) were tested for their reactivity against the merozoite surface proteins MSP1-19, MSP3 and AMA1 by ELISA. The antigens had been selected on the basis of cumulative evidence of their role in anti-malarial immunity. Additionally, reactivity against crude *P. falciparum* lysate was investigated. Purified IgG from these samples were furthermore tested in an invasion inhibition assay for their antiparasitic activity.

**Results:**

Significant intra- and inter- population variation of the reactivity of the samples to the tested antigens were found, as well as a significant positive correlation between MSP1-19 reactivity and invasion inhibition (p < 0.05). Interestingly, male donors showed a significantly higher antibody response to all tested antigens than their female counterparts. In vitro invasion inhibition assays comparing the purified antibodies from the donors from Ghana and Madagascar did not show any statistically significant difference. Although in vitro invasion inhibition increased with breadth of antibody response, the increase was not statistically significant.

**Conclusions:**

The findings support the fact that the development of semi-immunity to malaria is probably contingent on the development of antibodies to not only one, but a range of antigens and that invasion inhibition in immune adults may be a function of antibodies to various antigens. This supports strategies of vaccination including multicomponent vaccines as well as passive vaccination strategies with antibody cocktails.

## Background

Malaria is a tropical disease found in most African countries including Ghana and Madagascar, because the high temperature and humidity combined with the presence of stagnant water provide ideal breeding conditions for the female *Anopheles* mosquito, the vector. It is a leading cause of morbidity and mortality, particularly in children living in endemic regions, causing 124–283 million infections and approximately 584,000 deaths per annum with no signs of a significant decline [1]. In Ghana, malaria accounts for at least 20 % of child deaths, 40 % of admissions of children to hospital and more than 50 % of outpatients [2].

Effective malaria vaccines remain an elusive goal despite the availability of the *Plasmodium falciparum* genome sequence, which makes malaria one of the few remaining severe infectious childhood diseases without any efficient vaccine. This is caused by a combination of factors, including the multistage lifecycle of the parasite (each with stage-specific antigens), its genetic diversity, and an incomplete understanding of its immunopathology, resulting in a lack of immunological markers correlating with immunity.

Antigens expressed on the surface of asexual blood-stage malaria parasites are major targets for antibodies elicited by infection. These IgG antibodies prevent merozoite invasion of red blood cells, as well as opsonize parasitized red blood cells, and prevent cytoadherence. Thus, they form a major component of the defense against asexual blood-stage parasites and are therefore prime targets for vaccine development. Susceptibility to infection and episodes of disease decline in frequency and severity over time, but it is unclear which asexual blood-stage antigens are targets for this naturally acquired immunity. The most likely marker candidates include merozoite surface protein 1 (MSP1) and its C-terminal product, (MSP1–19), apical membrane antigen 1 (AMA1) and merozoite surface protein 3 (MSP3), reflecting cumulative evidence of their role in naturally-acquired immunity to malaria based on epidemiological studies in countries such as Myanmar [3], Tanzania [4], Ghana [5–7], Kenya [8], Mali [9] and Venezuela [10].

MSP1 is a large protein which is proteolytically processed into the subunits MSP1-83, MSP1-30, MSP1-38 and MSP1-42 [11–13]. The MSP1-42 fragment is processed in a further step into MSP1-19 and MSP1-33 during erythrocyte invasion, leaving only the C-terminal cleaving product MSP1-19 bound on the surface of the pathogen by a GPI-anchor.

AMA1 appears on the surface of merozoites when released from the micronemes and undergoes processing from an 83-kDa precursor into a 66-kDa mature protein that is also known to play an essential role in erythrocyte invasion, forming the tight junction with the protein Ron2L [14]. During invasion the surface protein AMA1-66 is further processed and AMA1-48 as well as AMA1-44 are released into the blood stream [15–17]. For the processing of both proteins MSP-1 and AMA1, the protein subtilisin-like protease 2 (SUB2, sheddase) is responsible [18].

Many individuals with naturally acquired immunity to malaria produce anti-MSP1-19 and anti-AMA1-66 antibodies that play a critical role in their immunity by inhibiting erythrocyte entry. There is a strong correlation between these antibody titers and the levels of protection against malaria in endemic regions [19].

MSP3 is a 48-kDa protein found on the surface of merozoites, which unlike the other candidates, was identified by studying the monocyte-dependent parasite-inhibition effect observed following the passive transfer of IgG from immune African adults into infected Thai children [20]. Epidemiological studies confirmed that protection is associated with cytophilic responses against MSP3 [3, 21–23].

The present study profiled the immune response to MSP1-19, AMA1 and MSP3 within and between two diverse populations, in the malaria-endemic regions of Ghana and Madagascar, focusing on the ability of plasma from such individuals to inhibit erythrocyte invasion.

## Methods

### Study design/study population and blood sample collection

Blood samples from a voluntary study population, randomly recruited in the local communities residing in Kumasi (Ghana) and Mahajanga (Madagascar) were taken in February and March 2010 (for Ghana) and January 2010 (for Madagascar). These time points correspond to the beginning of the rainy season in Ghana and the peak of the rainy season in Madagascar. Malaria transmission in Ghana is stable throughout the year and seasonal in Madagascar, with peak transmission during the rainy season [24, 25]. A total of 136 adults comprising 73 females and 63 males aged between 25 and 49 years were enrolled in this population-based cross-sectional immuno-epidemiological study. Basic information was gained after anamnesis and physical examination. Only healthy individuals who had been without self-reported clinical signs of malaria infection for at least 2 years in Ghana and at least 5 years in Madagascar were eligible for the study. Additionally, only people staying for the given time either in Ghana or Madagascar were admitted to the study. No pregnant or nursing mother, or individual with a known inflammatory condition or anemia, based on a general physical examination and a complete blood count at the time of blood collection, was included in the study. Written informed consent was obtained from all participants after the goals of the study had been carefully explained. Study participants were remunerated for the travel expenses to and from the study site. Ethical approval was obtained from the Committee on Human Research Publication and Ethics (CHRPE) of the Kwame Nkrumah University of Science and Technology and the National Ethics Committee of the Ministry of Health and Family Planning of Madagascar. Blood was collected into heparinized vacutainers, the plasma was prepared and stored at −20 °C.

### Antigens

Four different kinds of antigen preparations were used during the study. For measurement of reactivity against the AMA1 antigen, a mixture of the diversity covering variants (DiCo1-3) were used [26]. MSP1-19 (sequence from *P. falciparum* 3D7, PlasmoDB, #Pf3D7_090300, amino acids 1608-1702) was produced in HEK293T in a construct containing a murine Igκ signal peptide for protein secretion and a C-terminal His_6_-tag for protein purification by immobilized metal affinity chromatography (IMAC), basically as described before [27]. Two N-P-S/T motives had been mutated in order to prevent N-glycosylation. MSP3 (sequence from *P. falciparum* 3D7, PlasmoDB #Pf3D7_1035400, amino acids 25-354) was produced in tobacco plants (*Nicotiana benthamiana*) using a construct containing a murine IgG heavy chain signal peptide for protein secretion, a C-terminal His_6_-tag for protein purification by IMAC and C-terminal KDEL for retention in the ER as described before [28]. Four potential glycosylation sites had been removed by replacing the respective Asn by Ala. Lysates from *P. falciparum* 3D7 cultures were prepared as described before [29].

### Enzyme-linked immunosorbent assays

The plasma samples from the African blood donors, as well as a pool of two plasma samples from European blood donors, were analysed by enzyme-linked immunosorbent assay (ELISA) to detect IgGs specific for the recombinant malaria proteins AMA1, MSP1-19 and MSP3, as well as general reactivity against the *P. falciparum* lysate, similar to the methods described before with minor modifications [30]. Microtitre plates were coated with 50 ng of the recombinant proteins diluted to 1 μg/ml in phosphate-buffered saline (PBS) at pH 7.4. After incubation overnight at 4 °C, the plates were washed three times with PBS containing 0.05 % Tween-20 (PBST) and blocked with 2 % nonfat milk powder in PBS for 1 h at 37 °C. African serum dilutions from 1:200 to 1:6400 were prepared, and incubated for 1 h at 37 °C.

The plates were washed three times with PBST and bound IgG was detected with alkaline phosphatase conjugated goat anti-human IgG (1:5000 dilution in PBS, Thermo Scientific, Braunschweig, Germany) for 1 h at 37 °C. After five washing steps in PBST, the signal was developed using the substrate *p*-nitrophenyl phosphate (*p*NPP). Absorbance of the samples was recorded at 405 nm after 15 min.

Quantitative evaluation of the ELISA results were performed by calculation of the slopes of the linear range of the graphs obtained by the dilution *vs* the obtained absorbance. Values given are relative activity whereby the background reactivity of the non-immune European donor is equal to one. Positive reactivity was defined as reactivity superior to the European sample plus twice its standard deviation (SD). To account for the inter-plate variances, a pool of positive samples was used on each plate.

### Protein A affinity chromatography of plasma samples

To prevent nonspecific inhibition from heparin and other plasma components, the human antibodies were purified by Protein A affinity chromatography and tested for efficacy in invasion inhibition assays. Each 300 μl plasma sample was diluted such as to obtain 1 ml in PBS (pH 8.0) and passed over an equilibrated Protein A column by gravity flow. The flow-through was discarded and the column was washed with 35 column volumes of wash buffer. The antibodies were eluted in five column volumes of 0.1 M glycine (pH 2.5) and collected into 200 μl of 1 M Tris–HCl (pH 8.0) to neutralize the eluate and prevent denaturation. The eluates were concentrated to obtain the original antibody concentration by centrifugation at 300*g* for 15 min in MicroSep columns (Pall, Dreieich, Germany) and the concentrates were sterile filtered and subsequently used in the erythrocyte invasion inhibition assay after concentration determination by Bradford analysis and confirmation of integrity by SDS-PAGE.

### Invasion inhibition assay

*Plasmodium falciparum* 3D7 parasites were cultured in standardized parasite culture medium (RPMI 1640, 0.5 % Albumax II (Life Technologies, Carlsbad, CA, USA), sodium bicarbonate 1 g/l, glucose 2 g/l, hypoxanthine 0.028 g/l, gentamycin 0.2 g/l, pH 7.2) and synchronized using the sorbitol method twice at ring stage during two consecutive cycles, the last one 24 h before starting the assay, as described before [30]. Protein A-purified IgG fractions (final dilution 1:10, final concentration approx. 0.5 mg/ml), positive control rabbit anti-AMA1 (final concentration 6 mg/ml) were applied to synchronized *P. falciparum* schizonts (parasitaemia of 0.1 %/final haematocrit 5 %/total volume 50 µl) in 96-well round bottom plates. Cultures were kept at 37 °C for 96 h (two complete erythrocyte cycles). After this incubation time, parasite cultures were stained for 10 min using 0.1 % ethidium bromide, and subsequently analysed by flow cytometry using a BDFACSCanto II (BD Biosciences, Heidelberg, Germany).

### Statistical analysis

The results are presented as mean ± SD for normally distributed data and as means and 95 % Confidence Intervals (95 % CI) for non-normally distributed data. Comparisons were performed using the Kruskal–Wallis ANOVA tests and Dunn’s post-tests for selected multiple comparisons. In cases of comparison of two populations, Mann–Whitney U rank test was performed. Categorical data were analysed using Fisher’s exact test or χ^2^ (Chi squared) trend analysis. Spearman’s Rank Correlation Coefficient (rho) was used to test for the degree of association between test parameters. A p value of < 0.05 was considered to be statistically significant. P-values in figures are represented by asterisks (*, p < 0.05; **, p < 0.01; ***, p < 0.001; ****, p < 0.0001) All statistical analyses were carried out using GraphPad Prism v6.07 for Windows (GraphPad Software, San Diego, CA, USA).

## Results

### General characteristics of the study population stratified by country and gender

Among the 136 participants selected in this study, 78 (57.4 %) were from Ghana and 58 (42.6 %) were from Madagascar. Approximately half (46.3 %) of the studied population were males, with a mean age of 31.9 ± 6.6 years, which was lower than that of the total study population (33.6 ± 6.8 years). The mean age of the females in the study was 35.1 ± 6.8 years. The participants from Ghana were younger (p < 0.0001) compared to their Malagasy counterparts. A similar trend (p = 0.007) was observed when the male study subjects were compared to their female counterparts.

**Fig. 1:**
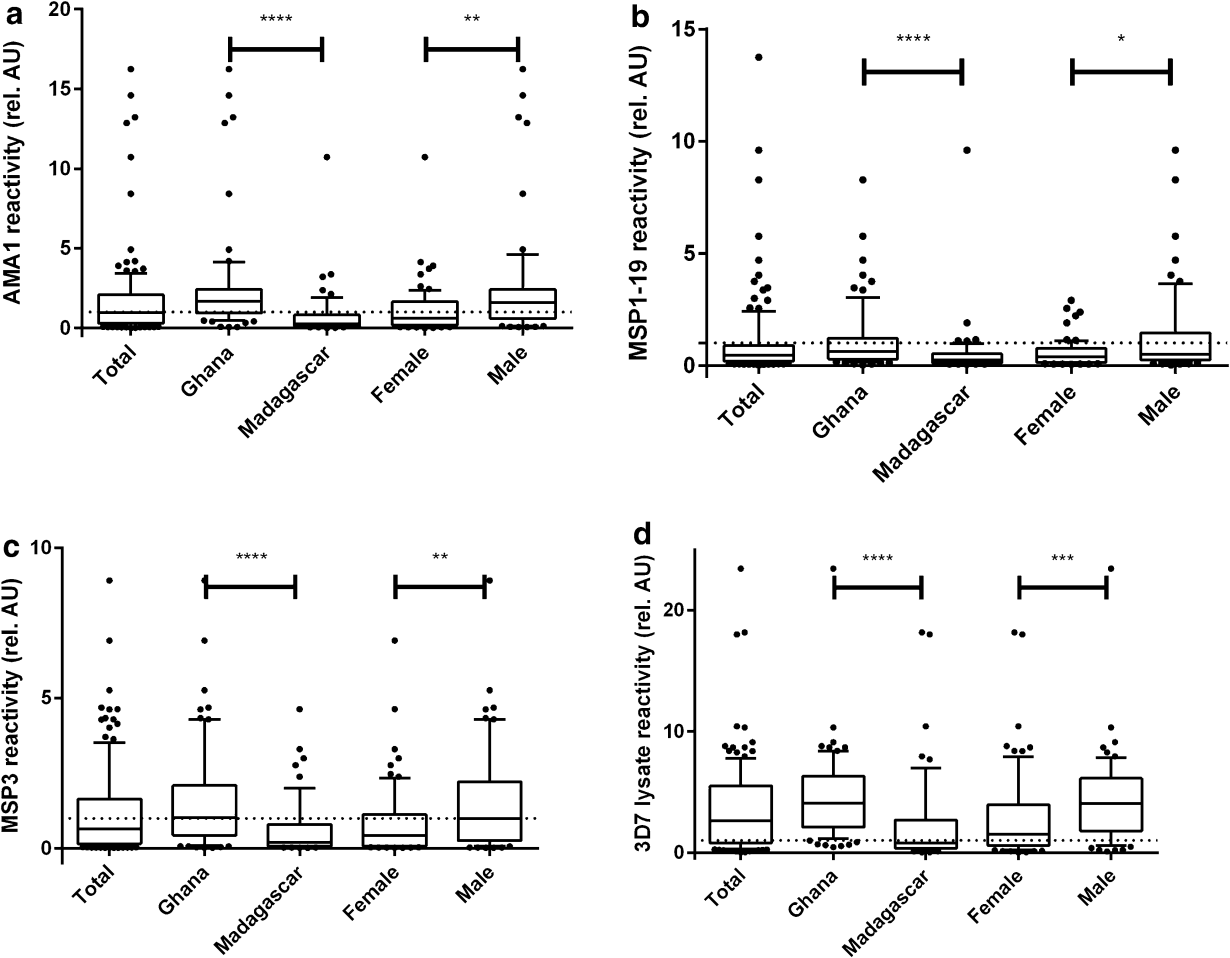
Antigen reactivity of plasma samples from the Ghanaian and Malagasy study population. Plasma samples were collected from 136 Ghanaian and Malagasy volunteers, and the respective reactivity towards the malarial antigens AMA-1 (**a**), MSP1-19 (**b**), MSP3 (**c**) and total 3D7 parasite lysate (**d**) quantified using standard ELISA towards 50 ng coated antigen each. As negative control, a pool of plasma samples from malaria-naïve European volunteers was used (*dashed line*). Sample reactivity was quantified after addition of goat anti-human IgG antibody (Thermo Scientific) at a dilution of 1:5000 and subsequent addition of pNPP. The arbitrary units on the *y-axis* are the sample reactivity divided by the reactivity of the negative control. For statistical analysis of the populations, the non-parametric Kruskal–Wallis and Dunn’s post test was used, *asterisks* indicate the level of statistical significance

The mean reactivity of the samples was highest against the 3D7 lysate followed by AMA1 and then MSP3, and lowest in MSP1-19. The same trend was observed when the study population was stratified by country and by gender (Fig. 1).

The mean reactivity and 95 % CI of the total study population against AMA1 was 1.7 (95 % CI: 1.3–2.2) (Fig. 1a). This was lower than the mean reactivity of the Ghanaian subjects 2.4 (95 % CI: 1.7–3.1) but higher than the one for the Malagasy subjects [0.8 (95 % CI: 0.4–1.2)], which means that the Ghanaian subjects showed a significantly higher reactivity to AMA1 than the Malagasy subjects (p < 0.0001). The mean reactivity of the male subjects in the study [2.4 (95 % CI: 1.6–3.3)] was significantly higher (p = 0.0012) than the one of the female subjects [1.1 (95 % CI: 0.8–1.5)].

Figure 1b, c and d show similar results for the other targets. The mean reactivity of the Ghanaian subjects was significantly higher than that of the Malagasy donors for MSP1-19 1.2 (95 % CI: 0.7–1.3) vs 0.5 (95 % CI: 0.2–0.9), (p < 0.0001), MSP3 1.6 (95 % CI: 0.9–1.5) vs 0.6 (95 % CI: 0.4–0.9) (p < 0.0001) and 3D7 lysate 4.5 (95 % CI: 3.8–5.3) *vs* 2.3 (95 % CI: 1.3–3.3) (p < 0.0001). The mean reactivity of the male subjects was also significantly higher than that of the female subjects for MSP1-19 1.3 (95 % CI: 0.8–1.7) vs 0.6 (95 % CI: 0.4–0.7) (p = 0.0442), MSP3 1.6 (95 % CI: 1.2–2.0) vs 0.8 (95 % CI: 0.6–1.1) (p = 0.0063) and 3D7 lysate 4.4 (95 % CI: 3.5–5.3) vs 2.9 (95 % CI: 2.0–3.7) (p = 0.0006).

### Antibody prevalence stratified by country and gender

**Fig. 2:**
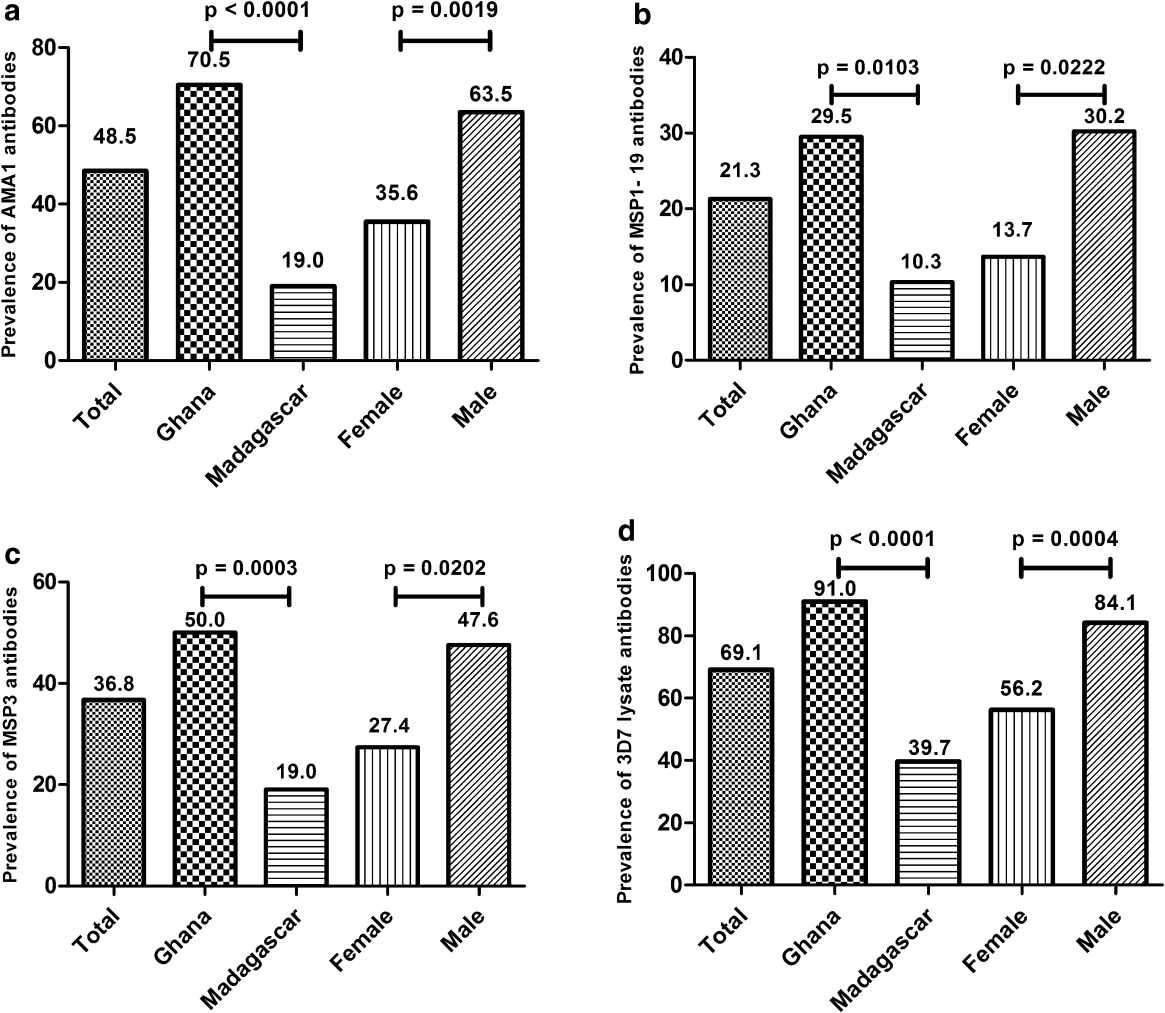
Prevalence of antibodies in plasma samples from the Ghanaian and Malagasy study population recognizing antigens **a** AMA1, **b** MSP1-19, **c** MSP3 and **d** 3D7 lysate stratified by country and gender. The antibody titers of the plasma samples were determined by ELISA. Positive reactivity was defined as reactivity higher than the respective negative control plus two times the value of the standard deviation of the negative control. Differences in the antibody prevalence were estimated by the Fisher’s exact test and in case of statistical significance corresponding p values are given

As shown in Fig. 2, antibodies against the 3D7 lysate were detected in 94 of the 136 subjects and were therefore the most prevalent (69.1 %). Antibodies against AMA1 were detected in 66 subjects (48.5 %), those against MSP3 were detected in 50 subjects (36.8 %) and those against MSP1-19 were found in 29 subjects (21.3 %). The Ghanaian subjects generally showed greater antibody prevalence than the Malagasy donors, and males showed greater antibody prevalence than the females (Fig. 2 and Table 1).

**Table 1:** Reactivity to AMA1, MSP1-19, MSP3 and 3D7 lysate stratified by country and gender

	Ghana		Madagascar		Kuskal–Wallis	Dunn‘s post test			
	Female (A)	Male (B)	Female (C)	Male (D)		p (A vs. B)	p (C vs. D)	P (A vs. C)	P (B vs. D)
Age (years, ± SD)	32.2 ± 7.4	29.2 ± 4.7	37.5 ± 5.1	38.7 ± 5.7	<0.0001	0.1617	>0.9999	0.001	<0.0001
AMA1 (AU)	1.6 (1.2–1.9)	3.0 (1.9–4.2)	0.7 (0.1–1.3)	0.9 (0.4–1.4)	<0.0001	0.5952	0.2926	<0.0001	0.0011
MSP1-19 (AU)	0.7 (0.5–1.0)	1.5 (0.9–2.0)	0.7 (0.0–1.4)	0.8 (−0.3–1.9)	0.0002	0.3937	>0.9999	0.0294	0.0043
MSP3 (AU)	1.0 (0.5–1.4)	2.0 (1.5–2.6)	1.0 (0.3–1.7)	0.9 (−0.2–2.1)	<0.0001	0.0141	>0.9999	0.2725	0.0002
3D7 lysate (AU)	3.5 (2.6–4.4)	5.2 (4.1–6.3)	2.3 (0.9–3.7)	2.4 (1.1–3.7)	<0.0001	0.0423	0.701	0.003	0.0005

Prevalence of antibodies to the variant antigens (%)						Chi square test			
N	33	45	40	18		p (A vs. B)	p (C vs. D)	p (A vs. C)	p (B vs. D)
AMA1	21 (63.6)	34 (75.6)	5 (12.5)	7 (38.9)		0.2541	0.0217	<0.0001	0.0058
MSP1-19	6 (18.2)	17 (37.8)	4 (10.0)	2 (11.1)		0.0608	0.8977	0.3116	0.0372
MSP3	11 (33.3)	28 (62.2)	9 (22.5)	3 (16.7)		0.0117	0.6119	0.3017	0.0011
3D7 lysate	27 (81.8)	44 (87.8)	14 (35)	9 (50.0)		0.0148	0.2800	<0.0001	<0.0001

Continuous data are presented as mean ± SD for the age and as means with 95 % confidence interval for the antigens reactivity and analyzed using the Kruskal–Wallis rank sum test and Dunn’s post test. Categorical data are presented as proportions and analyzed using a Chi square test

### Invasion inhibition results stratified by country and gender

**Fig. 3:**
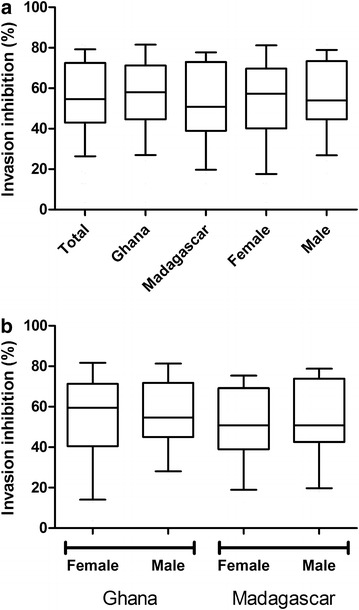
Box-plots of percentage invasion inhibition, stratified by country and gender. The invasion inhibition potential was estimated by a standard invasion inhibition assay using *Plasmodium falciparum* strain 3D7 and readout by flow cytometry after staining of the parasites with ethidium bromide. Groups were compared using non-parametric Kruskal–Wallis test. There was no statistically significant difference between the median invasion inhibition of the Ghanaians in comparison to the Malagasy, and the males in comparison to the females. The *lower* and *upper margins* of the *box* represent the 25th and 75th percentiles, and the *extended arms* represent the 10th and 90th percentiles. The median is shown as the *horizontal line* within each *box*

The invasion inhibition was estimated in vitro. The inhibitory effect of a positive control (rabbit anti-AMA1 polyclonal serum) was 84.8 ± 2 %. In the human study samples, despite the clear differences in antibody prevalence and reactivity, there was no significant difference in the efficacy of erythrocyte invasion inhibition when the study population was stratified by country and gender (Fig. 3). The median invasion inhibition was higher in the Ghanaians than in the Malagasy; these differences were, however, not statistically significant (Fig. 3a). It is interesting to note that although the male population showed a statistically significant higher antigen reactivity and prevalence over the females, there was no difference in invasion inhibition activity. When the participants were stratified by country and gender simultaneously to produce four comparative groups, there was again no statistically significant difference in the median invasion inhibition between Ghanaian males and females, and between Malagasy males and females (Fig. 3b).

### Correlation between reactivity and invasion inhibition

**Fig. 4:**
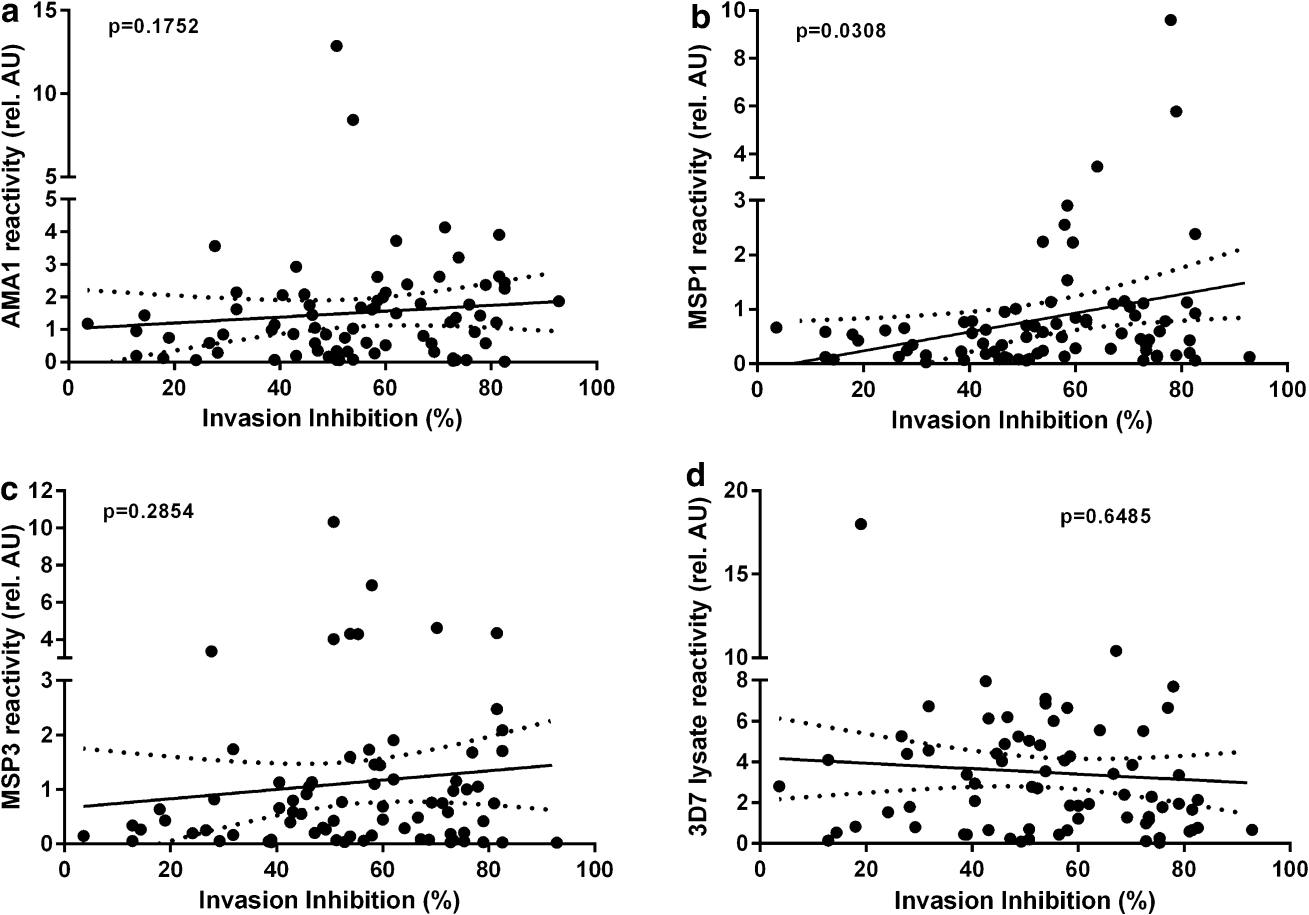
Linear regression between reactivity to the malarial antigens AMA1, MSP1-19, MSP3 and 3D7 lysate and erythrocyte invasion inhibition. Invasion inhibition activities of the antibodies, as well as specific antibody concentration in the plasma samples were estimated. The correlation of the data sets was estimated using the Spearman’s Rank Correlation Coefficient. The data shown represent the correlation of the invasion inhibition and **a** the concentration of AMA1-specific antibodies, **b** the concentration of MSP1-19-specific antibodies, **c** the concentration of MSP3-specific antibodies and **d** the concentration of 3D7 lysate-specific antibodies. The *lines* are the result of a linear fitting. 95 % confidence intervals of the linear fitting model are shown. Respective p values are given

Figure 4 shows a linear regression of reactivity and erythrocyte invasion inhibition for each antigen, which was carried out to test the use of reactivity to predict invasion inhibition efficacy. To test for the correlation of both variables, Spearman’s Rank Correlation Coefficient test was used. For every unit increase in MSP1-19 reactivity there was a significant increase of 57 % in erythrocyte invasion inhibition (p = 0.0308). For the other antigens, there was no correlation with the invasion inhibition tests detectable. All samples with a reactivity superior to 2 showed at least an in vitro invasion inhibition of 50 % (Fig. 4b). For the other antigens tested, no correlation of the corresponding reactivity in the tested samples and the potential to inhibit the invasion could be detected. The corresponding data and p values are shown in Fig. 4a, c and d.

### Invasion inhibition and breadth of the antibody response

**Fig. 5:**
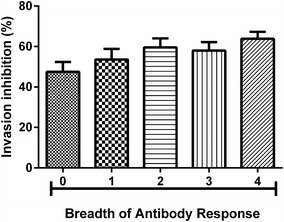
Relationship between invasion inhibition and the breadth of the antibody response in the Ghanaian and Malagasy study population. The breadth of the antibody response towards the antigens AMA1, MSP1-19, MSP3 and 3D7 lysate, each defined as a reactivity superior of more than two standard deviations above the negative control, was determined. To test for the dependence of the invasion inhibition on the breadth of antibody response, a Kruskal–Wallis test was performed

A Kruskal–Wallis test with a Dunn’s post test showed a gradual increase in the efficacy of erythrocyte invasion inhibition as the breadth of antibody response broadened (Fig. 5) although the trend was not statistically significant (p = 0.1444).

## Discussion

This population-based cross-sectional immuno-epidemiological study profiled the immune response to MSP1-19, AMA1, MSP3 and the *P. falciparum* 3D7 lysate within and between two diverse malaria endemic populations in Ghana and Madagascar with focus on the ability of plasma from such individuals to inhibit erythrocyte invasion. In this study, immune correlates of protection in two endemic regions were compared, in order to provide the basis for the distribution of the most antigen-specific immune responses in these areas. These study objectives were achieved by using ELISAs to measure total IgG antibody responses to MSP1-19, AMA1, MSP3 and the *P. falciparum* 3D7 lysate in the stratified study population. The prevalence of the antibodies was determined, and correlations between antibody reactivity, prevalence and erythrocyte invasion inhibition efficacy were investigated.

The relationship between the mean reactivity to MSP1-19, MSP3 and the 3D7 lysate antigens and age has been established in several studies [31–33]. However, most of these studies were done in infants, children and adolescents, whereas the present study focused on people with fully developed protective immune responses. This notwithstanding, the observations made in this study corroborate the findings of the earlier studies. On the other hand, the prevalence of antibodies was higher in Ghanaian males compared to Ghanaian females for both antibodies to MSP3 and to 3D7 lysate, in contrast to observations made elsewhere [10]. Nevertheless, this did not have an effect on the inhibitory potential of the purified polyclonal antibodies from these donors, giving a hint that the quantity of functional, (in vitro) neutralizing antibodies in both populations is similar. It is under debate whether the in vitro assays give a real hint for protection from clinical malaria in an epidemiological setting. Whereas Marsh et al. [34] did not find any relation of invasion inhibition and protection, Crompton et al. saw a significantly reduced risk of having a malaria attack in people with high in vitro invasion inhibition activity [35].

The reactivity of the samples to the three antigens (MSP1-19, AMA1 and MSP3) and one antigen mixture (3D7 lysate) considered in this study was related to the gender and ethnicity of the participants. This appears to be the first time in which all these antigens were examined in a single study to define the immune response in adults from malaria-endemic areas. Whereas Ghanaian men showed higher reactivity to all antigens in comparison to the Malagasy counterparts, Ghanaian women showed higher levels of reactivity against AMA1, MSP1-19 and 3D7 lysate compared to the Malagasy female counterparts (Table 1).

The variation in reactivity observed among the individuals may reflect a number of underlying factors, such as different exposure levels and transmission intensities across the study areas, the difference in the minimal time period since the last clinical malaria infection as reported during the recruitment, differences in host genetic factors, and differences in the predominant parasite strains in the tested areas. With regard to endemicity, Kumasi (Ashanti region, Ghana) represents a holoendemic region with year-round malaria transmission, whereas Mahajanga (Boeny region, Madagascar) is meso- to hyperendemic with a seasonal transmission pattern [36, 37]. The presence of other infectious diseases, whose epidemiology may be regional or gender-biased, may also prevent the development of antibodies against malarial antigens. There may also be erythrocytic heterogeneity among the stratified populations, resulting in the selection of particular parasite ligands that can mediate successful invasion [38].

A significant positive correlation between reactivity to MSP1-19 and invasion inhibition could be observed in the presented study, as well as a positive relationship between AMA1 and MSP3 reactivity and invasion inhibition albeit below the threshold of statistical significance. The clinical relevance for the specific in vitro invasion inhibition effect of antibodies which are specific for MSP1-19 and their protective effect from malaria infection has been reported before for a cohort from a highly endemic area in Kenya [39]. In this study, the upper quartile of donors had a significant protection from reinfection with the observation period of 10 weeks after parasite clearance. Nevertheless, they could not find a direct correlation of the total amounts of MSP1-19 antibodies and the concentration of inhibitory antibodies, which might be due to a high concentration of antibodies binding epitopes which are not or less relevant for direct invasion inhibition.

The mechanism of how AMA1 antibodies protect from malaria are regarded as similar to the protection offered by MSP1-19 antibodies. Both antibody types are capable of inhibiting the merozoite invasion directly by steric hindrance [40–42].

MSP3-specific antibodies are also described as being capable of inhibiting the invasion of merozoites into the erythrocytes directly [33, 43]. Generally, however, the mechanism of how MSP3-antibodies contribute to protection is regarded as being based on ADCI [30]. The purified human antibody fractions used in this study were polyclonal IgG likely directed at a whole spectrum of plasmodial antigens—as well as antigens completely unrelated to Plasmodium. Therefore, in order to pinpoint any effect of MSP3-specific IgG, it would be worth testing just this fraction of IgG of the semi-immune donors. In this study, this could not be performed due to the limited availability of plasma volume per sample.

The selection of the study population was based on the absence of a clinical malaria infection for at least 2 years. Immunity to malaria is thought to be short-lived, nevertheless, continuous exposure steadily boosts the immune response [44]. One explanation of the difference of antibody titers in male and female study participants could be a predicted difference in the respective entomological inoculation rates (EIR), but at least within the first year of life, some differences in the EIR just seem to have a limited effect on the antibody levels [45]. Within the first 2 years, children with lower exposure were found to have higher malaria-specific antibody levels, even though this relationship decreased with age [46]. The tendency for more males than females to congregate at night in many African communities due to cultural reasons might also explain the higher mean reactivity of the males in the study population. A study in Madagascar also found the interesting result that the incidence of malaria attacks during the high transmission season was higher in males than in females, giving a hint that they are more often infected and therefore might show higher parasite-specific antibody levels over time [25].

Despite the differences we found in polyclonal plasma IgG reactivity against AMA1, MSP1-19 and MSP3, a gender-related difference in the efficacy of invasion inhibition in vitro was not found in this study. The main aspect to consider here is that inhibition is likely brought about by a vast spectrum of different immunoglobulins—some of whose targets are yet to be identified—and not exclusively by AMA1-, MSP1-19- or MSP3-specific antibodies. Possibly, in women the protective antibody spectrum does differ as compared to men, but still unfolds the same inhibitory efficacy, at least in the in vitro assays.

A technical aspect of the measurement of the invasion inhibition is the potential loss of some subclass of IgG_3_ by the purification of the immunoglobulins by protein A chromatography. This might become especially important, when measuring the ADCI using monocytes, as here, IgG_3_ seems to play a crucial role [21].

Studies such as the one presented are the essential basis for the characterization of different populations in order to define the best qualified groups of individual donors for the isolation of human antibodies [28, 30]. Further studies should address (1) the effect of polyclonal IgG directed at one plasmodial antigen only, e.g., MSP3-specific IgG; (2) the incorporation of more plasmodial antigens in the analysis, e.g., PfRh4, PfRh5 or Ron2, the ligand of AMA1; (3) the effect of anti-plasmodial IgG besides mere binding, e.g., by incorporating immune effector cells such as neutrophilic granulocytes to assess secondary functions [29]. The immune response to the various antigens is relatively heterogeneous, indicating that in order to provide broad protection against the malaria infection; a vaccine should cover multiple malarial antigens.

## Conclusion

There is inter-population and inter-individual variation in the response of plasma samples to the malaria antigens tested. This is the first report of tests that (1) involves these selected antigens for the characterization of antibody levels in adults in two malaria endemic regions, and that (2) stratifies the study subjects on the basis of gender and geography; these may have implications on the design and distribution of a malaria vaccine.
